# An atlas of personality, emotion and behaviour

**DOI:** 10.1371/journal.pone.0227877

**Published:** 2020-01-21

**Authors:** Anthony E. D. Mobbs

**Affiliations:** Department of Psychology, Faculty of Human Sciences, Macquarie University, Sydney, New South Wales, Australia; Western Sydney University, AUSTRALIA

## Abstract

A novel two-dimensional matrix taxonomy, or atlas, of personality, emotion and behaviour is presented. The two dimensions of the atlas, affiliation and dominance, are demonstrated to have theoretical foundations in neurobiology and social psychology. Both dimensions are divided into five ordinal categories, creating a square matrix of 25 cells. A new catalogue of 20,669 English words descriptive of personality, emotion, behaviour, and power is also presented. The catalogue is more comprehensive than previous catalogues, and is novel in its inclusion of intrapersonal, group, and societal behaviours. All words in the catalogue were scored according to the atlas, facilitating visualisation in two dimensions. This enabled a contiguous and novel comparison of existing psychological taxonomies, as well as broader societal concepts such as leadership, ethics, and crime. Using the atlas, a novel psychological test is developed with improved sensitivity and specificity.

## Introduction

Since antiquity, humans have sought to identify a framework for understanding the whole person, encompassing personality, emotion and behaviour[1–3]. Raymond Cattell, who introduced factor analysis to personality research, originally trained as a chemist and stated that his lifelong ambition was to identify a model of personality with similar explanatory power as the periodic table of elements[4]. Taxonomies are systems of measuring or classifying phenomena that facilitate precise communication and common understanding. The law of parsimony states that ‘the simplest explanation of an event or observation is the preferred explanation’[5], or in other words, ‘Everything should be made as simple as possible, but not simpler.’[6]. Taxonomies may be said to be parsimonious if they precisely describe a broad range of phenomena with the minimum number of independent variables[7]. The characteristics of a parsimonious model of personality are well understood[3,8,9], yet no ‘periodic table’ or grand theory of the whole person currently exists[3,8]. There remains disagreement about the number or nature of personality factors[10] giving rise to the diverse variety of personality constructs[8]. Identifying a grand theory has been deemed one of the most important goals of personality research[11] with impact for both diagnostic and therapeutic understanding. Psychological connections have been observed between the concepts of emotion, behaviour and personality[12–19]. It was therefore hypothesised that a taxonomy may encompass all three concepts of emotion, behaviour and personality. A unifying taxonomy encompassing personality, emotions and behaviour would be more parsimonious than three separate taxonomies. It was hypothesised that the lexical approach could be utilised to identify an overarching taxonomy of personality, emotion and behaviour.

### Lexical analysis

The lexical hypothesis states that, ‘All aspects of human personality, which are or have been of importance, interest or utility, have already become recorded in the substance of language’[20] and, ‘When an idea is important, people are likely to have a word for it … the more important something is, … the more words there are likely to be’[21]. Lexical analysis is typically performed in two phases. Firstly, the words relevant to a topic are catalogued. Secondly, the catalogue is analysed to identify a parsimonious taxonomy that reduces complexity, simplifies categorisation and enhances communication. The completeness of the catalogue is desirable to ensure optimal selection of the form and parameterisation of the taxonomy. Conversely, an incomplete catalogue may lead to inappropriate selection of the taxonomic form or incorrect parameterisation.

Previous taxonomies of personality derived from lexical analysis, such as 16PF[22], HEXACO[23] and five-factor models[24] have largely focussed upon adjectival descriptors of personality. Verbs and nouns have largely been excluded[25], with notable exceptions[26,27], therefore leading to incomplete catalogues. In the context of lexical analysis, there are a range of verbs and nouns that are related to adjectival descriptors of personality traits. For example, verbs are used to describe interpersonal interactions (e.g. hit, hug, and harmonize) whereas abstract nouns may be used to describe emotions (e.g. hate, happiness and helplessness) and other nouns may be used to describe power (e.g. celebrity, chief, rich and poor).

### Interpersonal circumplex

For thousands of years, circles have been used in various ways to map the breadth of the human experience[28]. For instance, ancient Greek astrology divided the sky into twelve equal portions of a circle, and from that derived the star-signs which are still discussed in popular culture today. The Interpersonal Circumplex was born out of this tradition. In the early-mid twentieth century, the American psychoanalyst, Harry Stack Sullivan, began mapping theories of interpersonal dynamics in circular forms. One sketch from 1948 was similar to the subsequent Interpersonal Circumplex; it depicts two individuals in an interpersonal interaction, connected by one arc representing a disaffiliative force, and two arcs representing affiliative forces. After Sullivan’s death, the Kaiser Foundation Group, which included Timothy Leary, operationalised Sullivan’s concepts and were credited with discovering the circumplex. Leary continued developing the Interpersonal Circumplex, which gained international recognition through his seminal text, ‘The Interpersonal Diagnosis of Personality’[1]. The Interpersonal Circumplex became a watershed theory in personality psychology, and although it is not often used in contemporary application, it remains a foundational influence.

**Fig 1:**
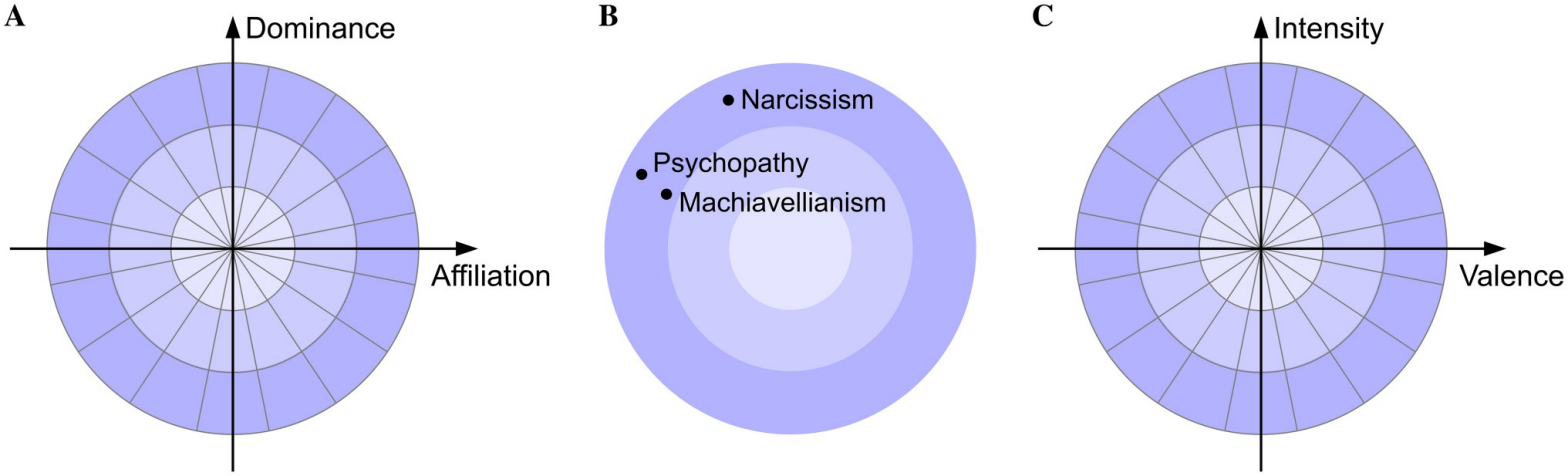
The Interpersonal Circumplex. (A) Interpersonal Circumplex used as a taxonomy of personality[1,30,45] with radially divided concentric circles superimposed over the two orthogonal dimensions of affiliation and dominance. (B) The Interpersonal Circumplex used to represent the Dark Triad[41]. (C) The Interpersonal Circumplex used as a taxonomy of emotion.

Interpersonal Circumplex taxonomies[1,29,30] are characterised by radially divided concentric circles superimposed over two orthogonal axes (Fig 1A). When used as a taxonomy of personality, affiliation and dominance have commonly been used as the orthogonal dimensions[1,31–34]. Other researchers have used a range of synonymic terms for affiliation and dominance including: agency/communion[35], getting-ahead/getting along[36], ambitious/agreeable[37], assertiveness/compassion[38], dominant/friendly[29,39] and domineering/nurturant[40]. The superimposed concentric circles measure intensity, with the least intensity at the origin and gaining intensity in proportion to the radius. The most extreme behaviours and personality descriptors are located on the perimeter of the outermost concentric circle. Many psychological constructs have previously been mapped onto Interpersonal Circumplex models; for example, Fig 1B shows the components of the Dark Triad mapped onto the Circumplex[41]. The circumplex has also been used as a taxonomy of emotion in which case the valence/affect and intensity/activation are often used as the orthogonal dimensions[42–44].

**Fig 2:**
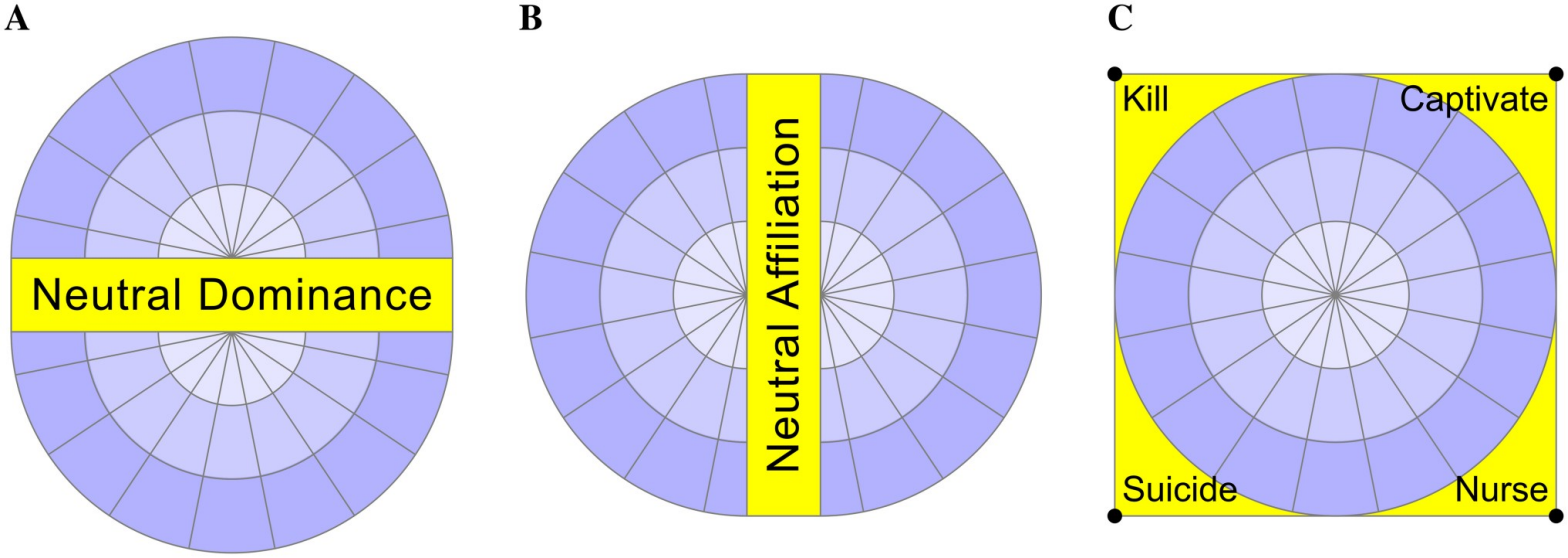
Deficiencies of the Interpersonal Circumplex. (A) Adominant behaviours (neutral dominance) not specifically identified on previous versions of the Interpersonal Circumplex, for example: ‘separate’, ‘gamble’ and ‘cooperate’. (B) Intrapersonal behaviours (neutral affiliation) not described on previous versions of the Interpersonal Circumplex[51] ‘innovate’, ‘learn’ and ‘stagnate’. (C) Maximally dominant and disaffiliative behaviours, such as killing, fall beyond the limits of the outermost concentric circle of the Interpersonal Circumplex.

Criticisms of the circumplex approach have included the apparent subjectivity of item placement[46], difficulty in operationalising due to overly numerical application[47], the superior performance of alternative taxonomies[48] and the inability to place personality disorders[49]. Proponents of the Interpersonal Circumplex have used methodological devices such as rotations in an apparent effort to overcome its inherent limitations[50]. In addition to these criticisms observed by others, three additional deficiencies of the Interpersonal Circumplex were identified in the present research. Firstly, that the constraints imposed by the use of concentric circles to measure intensity, impose correlation between otherwise orthogonal axes. Secondly, the Interpersonal Circumplex does not make allowance for behaviours and traits of neutral dominance (Fig 2A) or neutral affiliation (Fig 2B). Finally, Interpersonal Circumplex taxonomies are ambiguous as to the placement of extreme behaviours. For example, a behaviour that is both maximally dominant and maximally disaffiliative, such as killing, is unable to be mapped onto the Interpersonal Circumplex models with precision due to the concentricity constraint (Fig 2C).

The Abridged Big Five-Dimensional Circumplex (AB5C)[52,53] advances the Interpersonal Circumplex and measures many additional traits, thus overcoming some of the identified deficiencies. Although the Abridged Big Five-Dimensional Circumplex incorporates behaviours and traits of neutral affiliation and dominance, it retains the requirement for intensity to be measured by concentric circles, perpetuating the imposed correlation between axes and the ambiguous placement of extreme behaviours.

### Proposing a unifying taxonomy

The characteristics of a parsimonious taxonomy of personality have previously been described[3,8,9], four of which are:

1. **Comprehensive**: Encompassing all of what psychologists mean by ‘personality’[3,8].
2. **Synthetic**: Integrating knowledge of the various components of personality within a single coherent framework[3].
3. **Mechanistic**: Encompassing the biological basis of the mechanisms responsible for personality[3,8].
4. **Specific**: The dimensions of the taxonomy should be orthogonal and divisible into non-overlapping categories so that phenomena may be uniquely placed within the taxonomy[9].

No current taxonomy appears to satisfy all these criteria. Narrow taxonomies, such as the Dark Triad[41] are neither comprehensive nor synthetic, as they specifically limit their scope to a particular subset of behaviours (e.g. Psychopathy, Narcissism, and Machiavellianism). Factor taxonomies, such as 16PF[22], HEXACO[23] and five-factor models[24] are derived using dimensional reduction techniques such as principal components analysis or factorisation. Although there is clear evidence of heritability of personality characteristics[54], there is currently no accepted theory as to the neurological mechanisms that support the dimensions of factor models[55]. Other criticisms of five-factor models include the unexplained correlations between dimensions[24,56–60] and that factor models have been assessed as being insufficiently comprehensive[61]. For these reasons, both narrow and factor taxonomies were excluded from consideration as the foundation of a parsimonious taxonomy.

**Fig 3:**
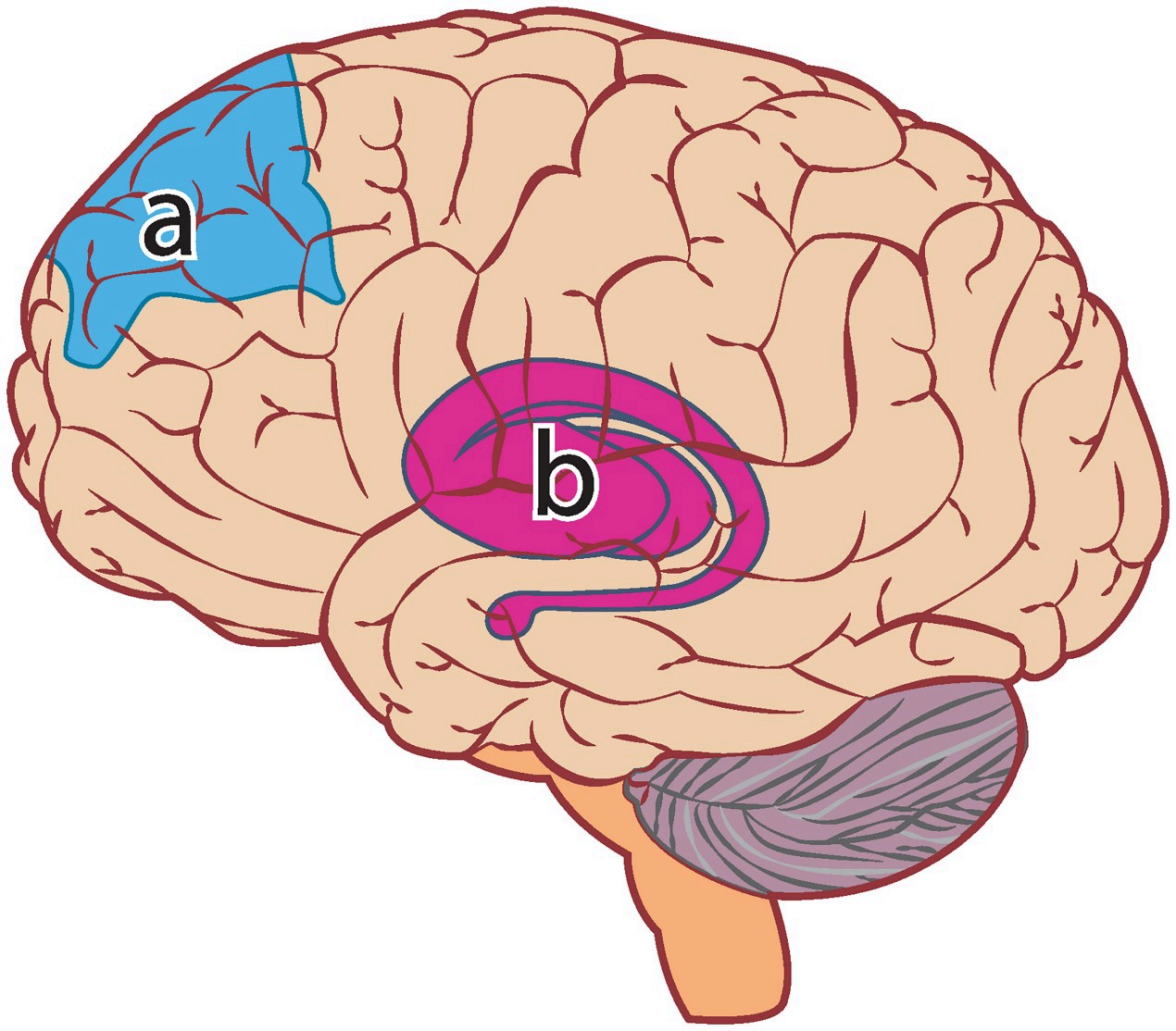
Areas associated with affiliation and dominance in the human brain. (A) The dorsolateral prefrontal cortex is related to dominance and submissiveness. (B) The Putamen is related to affiliation.

In contrast to five-factor models of personality, the dimensions of the Interpersonal Circumplex, affiliation and dominance, have strong biological support. Functional neuroimaging has identified independent neural pathways for affiliation and dominance (see Fig 3)[62]. These neural pathways have been identified in non-mammalian vertebrates[63] and are evident across five major vertebrate lineages of mammals, birds, reptiles, amphibians, and teleost fish[64]. The psychoactive hormones of oxytocin and testosterone have also been correlated with affiliation and dominance[65,66]. Additionally, the evolutionary bases of affiliation and dominance have been extensively investigated and established[67–69]. Given the comprehensive usage and biological basis for the selection of affiliation and dominance, we conclude that affiliation and dominance satisfy the criteria for being mechanistic and are therefore suitable candidates for the dimensions of a parsimonious taxonomy of personality.

To avoid ambiguity of trait placement and achieve the requisite level of specificity required for parsimony, two structural modifications to the Interpersonal Circumplex are proposed. Firstly, to overcome the imposed correlation between dimensions, it was proposed to remove the concentric circles. Therefore, behaviours and personality traits may be measured independently by reference to each orthogonal axis. Secondly, to allow for the categorisation of behaviours and traits of neutral dominance and affiliation, one of the ordinal categories for each dimension must be created to specifically allow for neutral behaviours. In keeping with the concept of neutrality, this category will be assigned the value of 0.

In order to categorise phenomena according to the taxonomy with specificity, each dimension must be divided into non-overlapping categories[9]. Ordinal categories are proposed that measure the enduring effect of behaviour on observers (Table 1). Behaviours that have negligible effect on observers were scored towards the centre of the scale. Behaviours that have an enduring impact on the observer were scored towards the extremity of the scale. Affiliation was scored as positive and disaffiliative behaviours as negative. Dominant behaviours were scored positive and submissive behaviours, negative.

**Table 1:** Temporal scale of enduring effect an actor’s behaviour has on observers.

	Affiliation	Dominance
**2**	The actor asserts, proffers, evokes or induces enduring affiliation with others	The actor asserts, proffers, evokes or induces enduring dominance over others
**1**	The actor asserts, proffers, evokes or induces transient affiliation with others	The actor asserts, proffers, evokes or induces transient dominance over others
**0**	The actor’s behaviour is neutral	The actor’s behaviour is neutral
**-1**	The actor asserts, proffers, evokes or induces transient disaffiliation with others	The actor asserts, proffers, evokes or induces transient submission to others
**-2**	The actor asserts, proffers, evokes or induces enduring disaffiliation with others	The actor asserts, proffers, evokes or induces enduring submission to others

An actor may attempt to assert, proffer, evoke or induce states of affiliation and/or dominance with respect to an observer; however, the response of the observer is inherently influenced by their perception of the actor’s behaviour/emotion. These perceptions may be dependent upon many factors, such as individual neurobiological variation in personality, cognitive states including attentional networks, power status, context, and the cultural milieu. For these reasons, the proposed classification scale does not specify or infer causal relations, rather, it identifies correlations rated typical of general cases.

Representative examples of the application of this table include:

1. Behaviours that are maximally disaffiliative and maximally dominant (-2,2) include ‘maim’, ‘attack’, as well as the absolute behaviour, ‘kill’. Observers of these behaviours may label the actor as ‘cruel’, ‘violent’ or ‘criminal’. Antecedent emotions to such behaviours and traits include ‘rage’, ‘anger’ or ‘wrath’.
2. Behaviours that are maximally disaffiliative and maximally submissive (-2,-2) include ‘self-harm’ and the absolute behaviour, ‘suicide’. Observers of these behaviours may label the actor as ‘dejected’, ‘morose’ or ‘melancholic’. Antecedent emotions to such behaviours and traits include ‘despair’, ‘emptiness’ or ‘futility’.
3. Behaviours that are maximally affiliative and maximally submissive (2,-2) include ‘worship’, ‘honour’ and ‘adore’, and the absolute behaviour, ‘martyrdom’. Observers of these behaviours may label the actor as ‘reverent’ or ‘devoted’. Antecedent emotions to such behaviours and traits include ‘love’, ‘veneration’ and ‘devotion’.
4. Behaviours that are maximally affiliative and maximally dominant (2,2) include ‘charm’, ‘excite’ and ‘inspire’. Observers of these behaviours may label the actor as ‘exuberant’, ‘dynamic’ or ‘charismatic’, and the absolute behaviour, ‘perfection’. Antecedent emotions to such behaviours and traits include ‘ecstasy’, ‘passion’ or ‘triumph’.
5. Behaviours that are of neutral dominance and affiliation (0,0) will be rarely noticed by either the actor or observer(s). These neutral emotions and behaviours include the awareness of our basic senses and involuntary behaviours such as ‘digestion’ and ‘respiration’.

**Fig 4:**
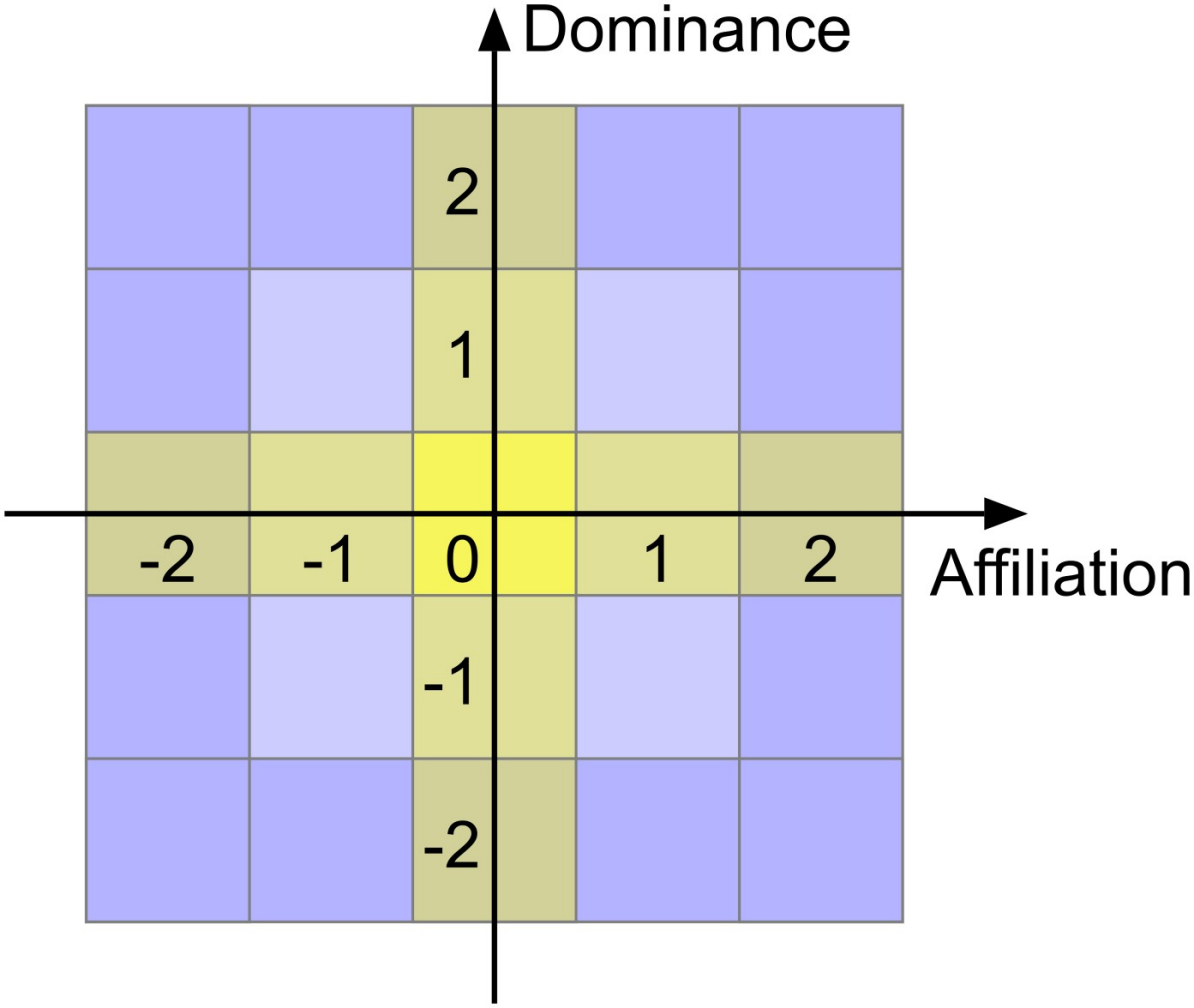
Atlas of personality, emotion and behaviour. Matrix taxonomy of personality created by ordinal division of the affiliation and dominance dimensions. Neutrally affiliative and dominant behaviours and traits are shown in yellow. Behaviours may be independently classified by reference to each orthogonal axis.

The atlas taxonomy addresses the deficiencies of the Interpersonal Circumplex, thus forming a parsimonious taxonomy of human personality. By removing the radial and concentric constraints and dividing each axis into ordinal divisions, a matrix structure is created, see Fig 4. The matrix’s standard taxonomic form[9] resembles other parsimonious taxonomies, such as the periodic table of elements and cartographic maps. This enables the full spectrum of interpersonal, intrapersonal, dominant, submissive and adominant behaviours to be precisely measured.

Studies 1 to 4 in the present research seek to confirm that the proposed atlas taxonomy is both comprehensive and synthetic. If so confirmed, the atlas taxonomy will be demonstrated to be comprehensive, synthetic, mechanistic and specific, and will therefore satisfy the criterion for being a parsimonious taxonomy of personality. Study 5 in the present research develops a pilot psychological test founded upon the atlas that may be used as the basis for future research using the atlas.

## Study 1

The objective of this Study was to catalogue all English language words descriptive of human interaction and emotion.

### Method

WordNet[70] was identified as a reputable lexical database for the English language developed within the Princeton University Department of Psychology. Multiple scans of the WordNet repository cataloguing all words descriptive of emotions, behaviour, personality and power. The Oxford and Merriam Webster thesauri[71,72] were used to identify synonyms and antonyms for all catalogued words. The synonyms and antonyms descriptive of emotions, behaviour, personality and power were added to the catalogue. This process was repeated until no further words were identified. The Oxford English Dictionary[71] was used to classify the part of speech for each word.

To achieve consistent categorisation, the four concepts were defined as follows: behaviour is an observable action (typically verbs), emotion is the perception of a neurological impulse that initiates behaviour (typically abstract nouns)[73], personality traits are descriptors of characteristic behaviours (typically adjectives), and power is the capacity to influence another, or the capacity to avoid being influenced by another (typically nouns)[74–77]. The concepts of power and the trait of dominance are often conflated, yet others have sought to differentiate these concepts[78]. Power has been observed to have a significant moderating effect on personality and emotion. Given the moderating effect of power on personality and emotion, the present research distinguished power from other concepts where possible.

## Results

20,669 words were identified as being descriptive of personality, emotion, behaviour and power. A summary of the words is shown in Table 2.

**Table 2:** Summary of words denoting personality, emotion and behaviour and power.

*Part of Speech*	*Domain*				
	Behaviour	Emotion	Personality	Power	Total
Adjectives	-	-	4,356	171	4,527
Idioms	3,675	532	1,915	464	6,586
Nouns	1,345	2,705	1,047	1,335	6,432
Verbs	3,124	-	-	-	3,124
**Total**	**8,144**	**3,237**	**7,318**	**1,970**	**20,669**

Examples of the 1,970 words descriptive of power, included ‘rich’, ‘poor’, ‘skilled’, ‘unskilled’, ‘employed’, ‘unemployed’, ‘king’ and ‘servant’.

### Discussion

Previous catalogues of adjectives are in the range of 1,710 to 4,500 words[27,79–81]. The limited size of previous catalogues casts doubt on the completeness of taxonomies derived from them. Reconciliations of the new catalogue were performed with previous catalogues where available. The reconciliations showed that previous catalogues included archaic words that are uncommon in modern dictionaries, such as ‘indeliberate’, ‘granousier’, ‘eremitic’ and ‘scientistic’. The reconciliation showed that currently popular words, such as ‘adaptable’, ‘charismatic’, ‘perfectionist’ and, ‘withdrawn’, had been omitted from earlier catalogues. This is demonstrative of the ability of the catalogue to be culturally sensitive. The existence of modern online word catalogues, dictionaries and thesauri greatly assisted the compilation of the catalogue, and thus formed the most comprehensive catalogue of English-language words in personality research to date. The procedures adopted, as well as the absolute number of words identified, formed a catalogue that was considered to be unbiased and sufficient for the purposes of identifying a comprehensive taxonomy.

Despite the comprehensiveness of the catalogue, without replication, it is possible that important descriptors of personality, emotion or behaviour may have been overlooked.

## Study 2

The objective of this study was: firstly, to confirm that the words in the catalogue can be classified according to the atlas matrix taxonomy, and secondly, to score a subset of up to 20% of the catalogued words.

### Method

A Delphi approach was utilised to obtain a reliable consensus of opinions of a small group of health professionals[82]. The Delphi approach requires a group of experts to independently record their professional opinion and then achieve consensus through discussion. A cognitive and behavioural neurologist and three registered clinical psychologists assisted as linguistic judges. It was determined that a relatively small subset of the catalogue could be manually scored and therefore a five phase Delphi approach with sampling was used.

**Phase one**: To ensure the Delphi process covered all of the 25 cells of the atlas, the author performed an initial scoring of the entire catalogue. From this initial scoring, 10 nouns, 10 verbs and 10 adjectives were selected from each of the 25 cells of the atlas (750 words in total).

**Phase two**: Without being informed of the scoring performed in Phase one, the judges were asked to independently score each word. The judges then discussed their scores, during which, the judges were encouraged to revise their individual scoring until consensus was achieved.

**Phase three**: Synonyms of words scored in Phase two were identified from which were selected 35 nouns, 35 verbs and 35 adjectives from each cell (1,925 words in total). These word lists were collectively discussed by the judges. The scoring of each word was reviewed by exception until consensus was achieved. It was observed in this phase that the judges referred to synonyms and antonyms of other words selected to achieve a level of consistency in their scoring. Subsequent to the group process, the author and neurologist rescored the remainder of the catalogue.

**Phase four**: The author selected 1,620 antonymic word pairs. The word pairs consisted of words made opposite by prefix or suffix, such as ‘observant/unobservant’, ‘engaged/unengaged’ and ‘merciless/merciful’, and word pairs identified as antonyms in either or both of the reference thesauri. If both words had previously been scored as diametrically opposite in earlier phases, the scoring was retained. If both words had not been previously scored as diametrically opposite, a manual revision of their scoring was performed to achieve exact opposite scoring. These word pairs were then independently reviewed by three judges. In cases where at least two of the three judges had assigned identical scoring to word pairs, these word pairs were considered to be archetypal. The remaining words scored by the judges in Phase three were also deemed to be archetypal.

**Phase five**: The author reviewed the scored archetypal words and made minor amendments to ensure that synonyms, conjugates and inflections were proximately located where appropriate. The judges then performed an overarching review of a summary of 1,800 archetypal words until consensus was achieved by all judges (see S1 Table).

It was identified that there are examples of English language words that can be used in multiple contexts. In these situations, the words are usually classified as different parts of speech. For example, ‘bully’, ‘calm’ or ‘tidy’ all of which can all be descriptive of a behaviour (verb) or personality trait (adjective). In these instances, the words were categorised by the judging panel in the same cell irrespective of the context. An example of a word that can be used in different contexts but would be categorised in different cells is ‘humble’. ‘Humble’ used as an adjective was scored by the reviewers as having affiliation of 1 and dominance of -2. When used as a verb, ‘humble’ was scored as having affiliation of -2 and dominance of 1. When such instances were detected, the word was excluded as being candidate archetypal words. However, due to there being relatively few words in the cell (1,-2) and the word ‘humble’ was retained as an archetypal word for this cell.

### Results

Of the 1,620 antonymically opposite word pairs, 27 were rejected by two or more judges leaving 1,593 word pairs remaining. Of the remaining 1,593 word pairs, 150 were opposite due to a prefix or suffix, 711 were identified as antonyms in both reference thesauri and 731 were identified as antonyms in one of the reference thesauri. Consensus amongst the four judges was achieved for 96% of word pairs, with a single dissenting judge on 4% of word pairs. The near complete consensus was viewed as confirming that the catalogued words can be successfully classified using the atlas. Table 3 shows an example of a personality trait, emotion and behaviour for each cell in the atlas taxonomy.

**Table 3:** Example personality traits, emotions and behaviour applicable to each cell in the atlas.

		Affiliation				
Dominance	Domain	-2	-1	0	1	2
**2**	Emotion	hate	arrogance	enthusiasm	courage	euphoria
	Behaviour	attack	defy	pioneer	advance	captivate
	Personality	cruel	arrogant	energetic	brave	charismatic
**1**	Emotion	contempt	frustration	interest	confidence	happiness
	Behaviour	slander	argue	action	negotiate	laugh
	Personality	nasty	inflexible	efficient	confident	joyful
**0**	Emotion	detachment	instability	consciousness	stability	harmony
	Behaviour	dissociate	neglect	sense	attend	attach
	Personality	unfriendly	vague	alive	clear	friendly
**-1**	Emotion	sadness	anxiety	disinterest	appreciation	admiration
	Behaviour	lament	complain	inaction	relent	endorse
	Personality	joyless	worried	inefficient	flexible	nice
**-2**	Emotion	dread	cowardice	fatigue	humility	love
	Behaviour	deflate	surrender	stagnate	obey	nurse
	Personality	morose	coward	apathetic	humble	tender

Colouration applied to assist interpretation. See S1 Table for an expanded version of this table with 25 words for each combination of affiliation, dominance and domain.

### Discussion

The thesauri did not frequently identify synonymic associations between the words descriptive of personality, emotion and behaviour; for example, the words ‘kill’ (behaviour) and ‘murderer’ (personality) were not synonymously related. The reference thesauri did however nominate ‘killer’ (personality) and ‘murderer’ (personality) as synonyms. ‘Kill’ and ‘killer’ can be linked by virtue of having the same linguistic stem. The linking of stem words was performed manually in the present research; however, it could be automated in future. By supplementing the thesauri derived synonyms with manually linked stem words, a robust association between personality, emotions and behaviours was achieved.

Some emotions are known to give rise to physiological changes[83], such as happiness, love, pride, anger, fear, anxiety, shame, sadness, depression, disgust, contempt, and envy. It was noted that these emotions are located on the outer edge of the atlas. Strong emotions promote high levels of arousal, and the associated physiological changes are thought to lead to an evolutionary advantage by creating a state of readiness for action[84,85]. Conversely, emotions and behaviours at the centre of the atlas are largely occult or involuntary without any sense of urgency. Emotional intensity and readiness for action appear to be correlated concepts, and future analysis with the aid of the atlas may establish causality between these concepts.

Historically, it has been acknowledged that relationships exist between personality, emotions and behaviour, although the nature of these relationships is yet unclear. The present research has illustrated the linguistic associations between the catalogued words. Building upon this, future research may use the atlas as a tool to clarify the nature of the causal relationships between personality, emotion and behaviour.

**Fig 5:**
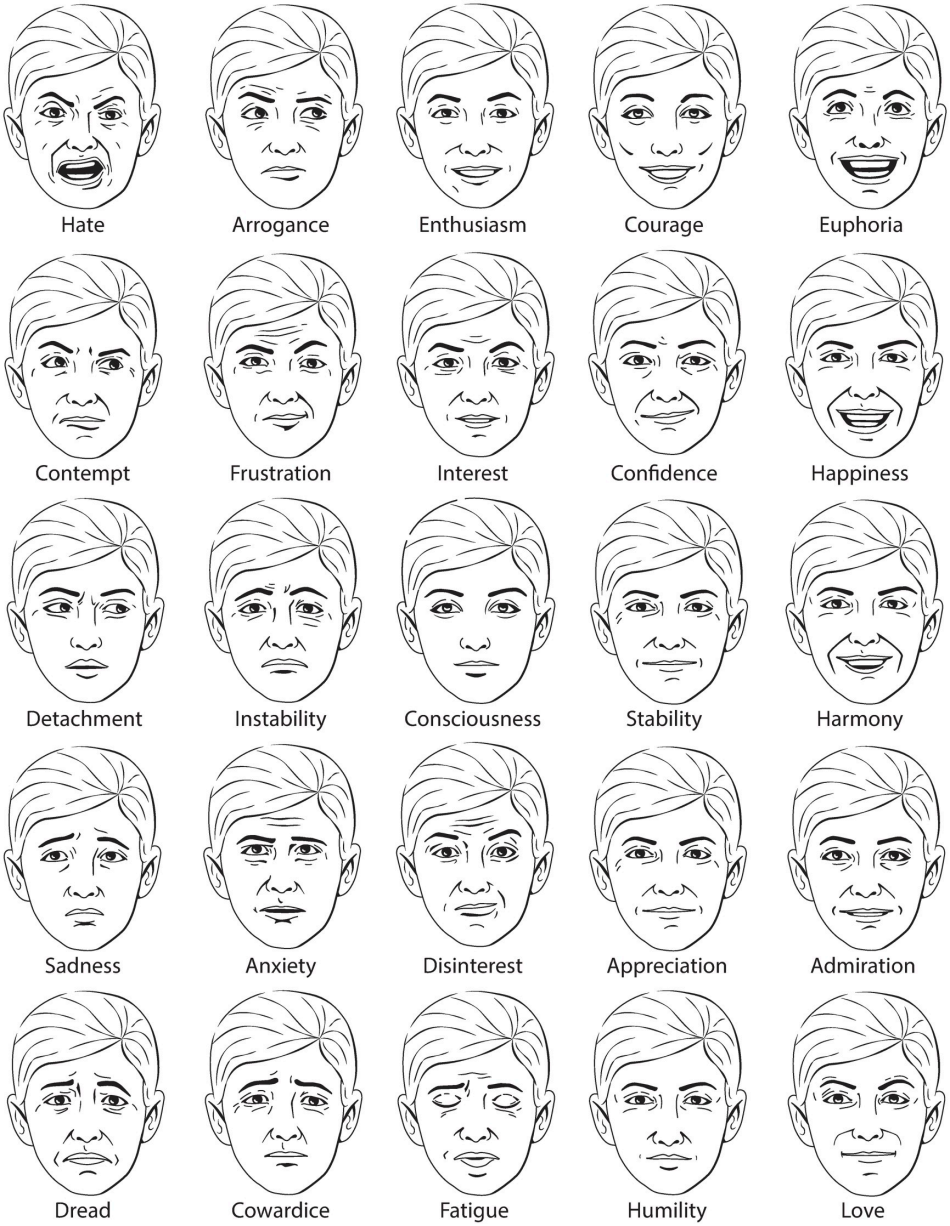
Artist's impression of emotional expressions according to the atlas.

**Fig 6:**
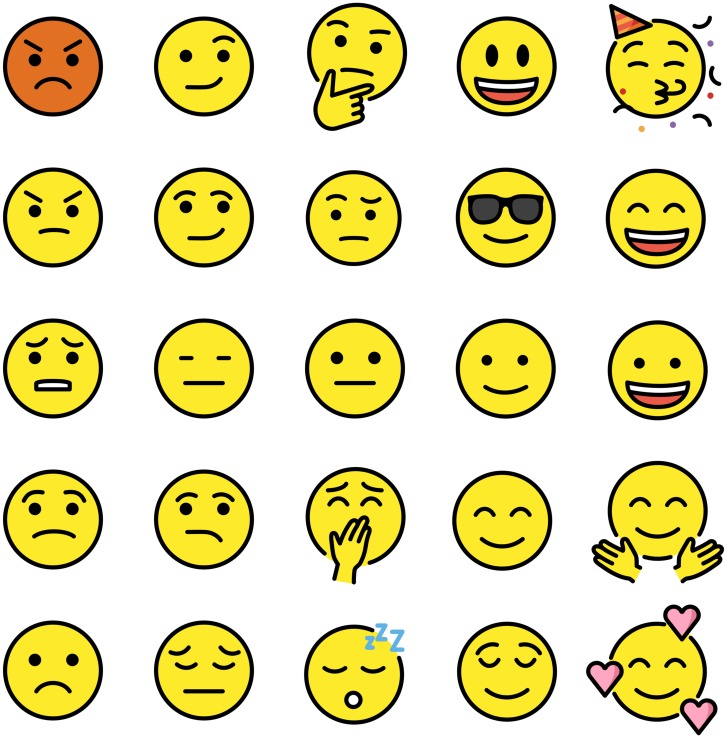
Emoji arranged according to the atlas. Images copyright https://openmoji.org/ distributed in accordance with Creative Commons Attribution-Share Alike licence (CC BY-SA 4.0).

Extensive research has been conducted on the human and non-human ability to recognise emotion through facial expression[18,19,86–88]. Several studies have used the Interpersonal Circumplex as a taxonomy for arranging the biological spectrum of facial expressions[34,89–91]. The atlas’ inclusion of emotions prompted the categorisation of facial expressions according to the atlas. An artist's impression of the emotions represented in Table 3 is shown in Fig 5. Emoji, which have become ubiquitous forms of electronic communication, have been arranged according to the atlas as shown in Fig 6. The facial expressions shown in Fig 5 demonstrate smooth gradients of expression according to the orthogonal dimensions of affiliation and dominance. The ability of the atlas to represent facial expressions lends support to efficacy of the two-dimensional model of personality and emotion, as well as the selection of affiliation and dominance as the two orthogonal dimensions.

A limitation of this study is that a large number of words were categorised by a small number of judges and that only 20% of the catalogue was manually scored. These limitations give rise to the possibility of bias. These limitations may be overcome by independent replication with additional judges and greater sample sizes.

## Study 3

The objective of this Study was to automate the scoring of words in the catalogue using a spring-based[92] network analysis.

### Method

Network theory has been used to visualise and analyse related concepts, including affiliation and dominance. Network theory has previously been applied to both psychological [93,94] and lexical [21,95,96] concepts. Networks consist of ‘nodes’ joined together by ‘edges’. In some network visualisations, such as transport networks, the stations (nodes) are connected, for example by tracks or roads (edges). For such networks, the location of the nodes and edges are physically fixed, and the network visualisation serves as a representation to assist commuters expeditiously travel between locations. Social networks are based on the connection of individual users (nodes) within a relationship network (edges). Networks may be concisely visualised by allowing the nodes to move freely on a two-dimensional surface. Nodes are joined together by edges that either attract or repel other connected nodes.

The equilibrium position of the nodes is determined when the combined attracting and repelling forces are minimised. When equilibrium is achieved, nodes connected by an attracting force are often proximate and nodes joined by a repelling force are normally distant from each other. A hybrid visualisation of networks is one in which some nodes are fixed and other nodes move freely. The attracting and repelling forces of each edge are typically modelled using the physical forces of Maxwell's equations of electromagnetic fields or Hooke's Law of springs[92]. Network representations of related words have been demonstrated by connecting words (nodes) by their synonymic and antonymic (edges) relationships[97,98].

In this Study, a network approach was used to categorise the affiliation and dominance for words not previously scored in Study 2, totalling approximately 80% of catalogue (nodes). Edges representing synonymic links were modelled as an attracting force and antonymic links modelled as repelling forces. The use of Hooke’s Law to model the attracting and repelling forces ensured that synonyms were closely proximate and antonyms were disparate when visualised using the atlas.

A Python computer program was developed to implement the following steps:

1. The synonyms and antonyms for all catalogued words were obtained from the Oxford and Merriam-Webster thesauri[71,72].
2. First iteration
  - For unscored words in the catalogue with 100% (threshold) of their synonyms previously unscored, the equilibrium position was calculated such that the forces of attraction between synonymic words and repulsion between antonymic words were minimised. Hooke’s Law was used to calculate the forces of attraction and repulsion. Hooke’s Law states that the force needed to extend or compress a spring is proportional to the extension or compression from the resting position. Synonyms that are distant from each other will experience a strong force of attraction whereas synonyms that are close together will experience no force. Conversely, antonyms that are close together will experience a strong repulsive force and antonyms that are distant from each other will experience no repulsive force.
  - Step a. was repeated by successively reducing the threshold (initially set at 100%) by 1% until all words in the catalogue were scored.
3. Subsequent iterations
  - The equilibrium position for each word in the catalogue was re-calculated in alphabetical order using the location of previously modified word scoring. This process allowed each word to freely move in the atlas, until equilibrium was reached and the words ceased to move.
  - This process was repeated until all words achieved their equilibrium position and no further word movements occurred.

### Results

A total of 543,830 synonymic pairs and 97,394 antonymic pairs were identified between words in the catalogue. Eight iterations of Step 3. were required until equilibrium was achieved. Table 4 shows the number of words within each cell of the atlas.

**Table 4:** Frequency of words in each cell of the atlas.

*domain*	*dominance*	*affiliation*					Total
		-2	-1	0	1	2	
behaviour	2	449	142	79	66	67	803
	1	343	992	1,514	497	99	3,445
	0	132	1,108	30	1,483	175	2,928
	-1	58	91	164	246	70	629
	-2	32	55	40	52	109	288
emotion	2	135	133	78	60	74	480
	1	189	318	256	169	58	990
	0	83	410	7	282	168	950
	-1	66	243	72	60	73	514
	-2	51	58	47	43	85	284
personality	2	220	279	133	229	79	940
	1	492	755	525	342	108	2,222
	0	267	1,390	10	816	177	2,660
	-1	127	227	374	84	108	920
	-2	39	102	183	115	80	519
Total		2,683	6,303	3,512	4,544	1,530	18,572

A qualitative review of the word placement by the spring-based network method confirmed that the method accurately placed the majority of words.

### Discussion

The spring-based network approach was able to efficiently score all words in the catalogue not previously scored in Study 2. A review of the scored words suggested that the process was accurate and satisfactory for the purpose of analysing existing psychological and social constructs.

## Study 4

The objective of this Study was to visualise in two dimensions a wide range of existing psychological, psychiatric and social constructs using the atlas.

### Method

A benefit of the atlas is that it is two-dimensional, consequently, a wide range of existing visualisation tools are readily available. One such method of visualisation is based on kernel density estimates[99,100]. Kernel density estimate plots were selected as they may easily visualise the relative density of items in particular regions of the atlas as compared to other regions.

The DSM-5 and ICD-11 were selected as the foremost texts on psychiatric disorders. The five-factor model and Dark Triad were selected as representative personality scales. Leadership, ethics and criminality were selected as important social constructs.

A Python library was developed that processes a list of words. The words were then mapped to their affiliation and dominance scores obtained in Studies 2 and 3. For example, the word ‘ordinary’ was scored with affiliation of 1 and dominance of 0, or (1,0), similarly ‘kill’ is translated to (-2,2) and ‘love’ (2,-2). Points on the atlas were then visualised in two-dimensions using the matplotlib Python library[101].

For each personality construct, a clinical psychologist assigned the most representative single word adjective to each question constituting the construct. For example, the clinical psychologist assigned the word ‘extroverted’ as being the most appropriate single word adjective to the question ‘I am the life of the party’. The DSM-5 and ICD-11 constructs were defined in terms of single word personality traits, emotions and behaviour, and were subsequently compiled into a list for each construct. For good and bad leadership behaviours, leading texts[102–104] were reviewed and the personality traits, emotions and behaviours were catalogued. For criminal behaviours, the criminal codes of several jurisdictions[105–110] were reviewed and the associated behaviours catalogued. For the concepts of good, bad, ethical and unethical, the synonyms in the reference thesauri[71,72] were catalogued for each term.

### Results

The results of this Study are visualised in Fig 6.

### Discussion

**Fig 7:**
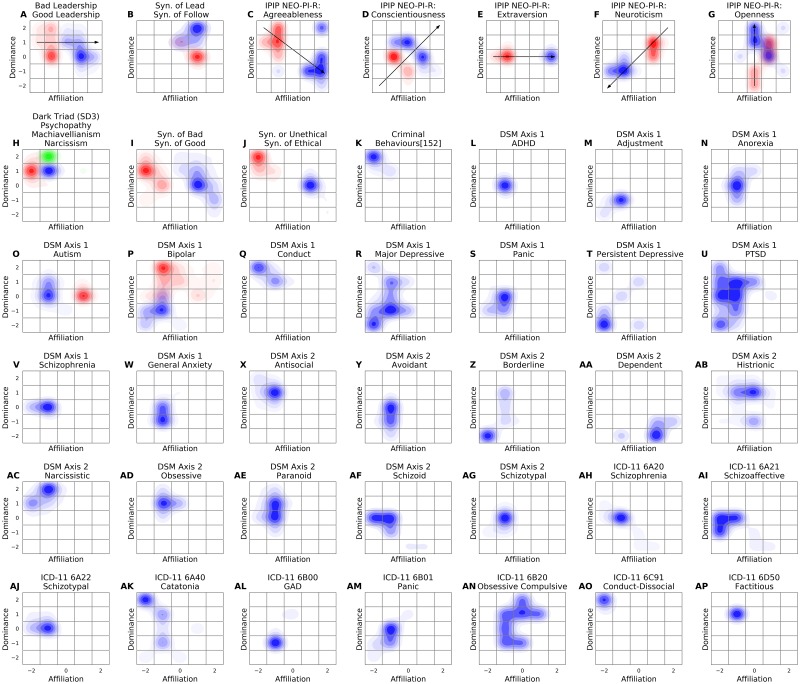
Visualisation of social and psychological constructs and personality disorders. (A) Good leadership (Blue) and bad leadership (Red)[102–104]. (B) Synonyms of Lead (Blue) and Follow (Red). (C-G) IPIP-NEO-120 Five Factor Model[111,112]. Positive valence (Blue), Negative valence (Red). (H) Dark Triad[41]. Narcissism (Green), Machiavellianism (Blue), Psychopathy (Red). (I) Synonyms of Good (Blue) and Bad (Red). (J) Synonyms of Ethical (Blue) and Unethical (Red). (K) Criminal behaviours[105–110] DSM-5 personality disorders[113]. Positive valence (Blue), Negative valence (Red). (AH-AP) ICD-11 Mental, behavioural or neurodevelopmental disorders[114].

It was demonstrated that the atlas can be used to visualise a range of important psychological and social taxonomies (Fig 7). The Dark Triad[41] was visualised by its authors using the Interpersonal Circumplex (Fig 1B). The similarity of the representation between the circumplex and atlas (Fig 7H) suggests convergent validity to the atlas in this instance. Other examples of convergence include psychopathy (Fig 7H), agreeableness (Fig 7C—negative valence), DSM-5 conduct disorder (Fig 7Q), ICD-11 Conduct-dissocial Disorder (Fig 7AO) and criminality (Fig 7AO) which converge in cell (-2,2) of the taxonomy. Similarly, the related concepts of good (Fig 7I), ethical behaviours (Fig 7J) and agreeableness (Fig 7C—positive valence) converge in the cells adjacent to (2,0). These findings match intuitive expectations about the co-location of certain constructs, and provide preliminary evidence for the convergent validity of the atlas.

The atlas was able to visually demonstrate and differentiate the poles of multi-pole concepts, such as concepts defined by poles of positive and negative valence. For example, the subsidiary concepts constituting the Dark Triad (Fig 7H), Autism (Fig 7O), Bipolar (Fig 7P), Five Factor Models (Fig 7C–7G) and social concepts such as leadership (Fig 7A and 7B) and ethics (Fig 7I and 7J). Of these multi-pole concepts, the visual delineation was least distinct between the two poles of openness (Fig 7G). Social concepts such as good and bad leadership (Fig 7A), leading and following (Fig 7B), good and bad (Fig 7I), and ethical/unethical (Fig 7J) do not appear to have been previously visualised using the Interpersonal Circumplex, yet were easily plotted and differentiated when visualised using the atlas. This may have implications for the practical application of the taxonomy to group and cultural dynamics.

The observed correlations between the five factors of five-factor models has hitherto evaded theoretical explanation[56,60,115,116]. However, the representation of the vectors facilitated by the atlas appears to provide a basis for understanding the observed correlations. The correlation between vectors may be calculated by determining the cosine of the angle between vectors at the point of intersection[117]. For example, neuroticism has been consistently observed to be negatively correlated with the other factors of five-factor models, which is consistent with the calculated correlation using the vector approach. Further confirmation of this result would represent strong evidence of there being two fundamental dimensions of personality.

The proposed atlas introduces a novel approach to visualising psychological and social concepts. There are, however, several hundred existing psychological constructs in existence and one limitation of this study is that only a small number have been visualised. The atlas could be further validated by visualising additional psychological constructs.

## Study 5

The purpose of this Study was to develop a pilot psychological test that may form the basis of future research based on the atlas.

### Method

To be comprehensive, it was determined that the psychological test must ask questions from all cells of the atlas. However, the centre cell of the taxonomy (0,0) identifies basic functions of living, such as occult emotions and involuntary and reflexive behaviours. Measuring involuntary and reflexive behaviours is unlikely to be of interest to individuals or personality researchers, and therefore the (0,0) cell was excluded from the test. After excluding the centre cell, 24 cells remained. It was determined that a single question could compare two cells simultaneously by asking dipole questions, where each option is sourced from a different cell, for example ‘Are you more often happy or unhappy?’. From this it was inferred that the minimum number of questions required for the proposed psychological test was 12.

Sensitivity and specificity are statistical measures often used to assess the performance of binary classification tests, and were presently used to determine the optimal configuration of the 12 dipole questions. The concept of sensitivity measures the proportion of correctly identified positives, and specificity measures the proportion of correctly identified negatives. To achieve high levels of sensitivity and specificity, a parsimonious test must ask questions that maximally distinguish the concepts under consideration. Greater distinction was hypothesised for antonymic binary choice questions as opposed to near synonymic binary choice questions. For example, antonymic binary choice questions such as ‘Are you usually friendly or unfriendly?’ are likely to have greater sensitivity and specificity when compared with near synonymic binary choice questions such as, ‘Are you usually friendly or polite?’. Therefore, the 12 dipole questions were restricted to antonymic binary choices.

In a matrix of 24 cells, there are _24_P_24_ ≈ 10^23^ permutations of binary questions that could be asked of the respondent. It is not possible with modern computational techniques to test all 10^23^ permutations in order to identify the combinations that maximise the overall contrast, therefore, a simulation and alpha-beta pruning[118] approach was used to determine which combinations maximise the average distance between the possible antonymic binary pairs.

Three measures were developed to compare the efficacy of various psychological tests and constructs. The first measure was termed ‘Completeness’ and defined as the proportion of cells in the atlas at which one side of a dipole was located. Tests with higher levels of completeness are likely to be efficacious for a diverse range of diagnostic applications. The second measure was termed ‘Diffusion’ and defined as the proportion of questions in the test that are represented by the atlas outside of the modal cell containing one side of the dipole. Tests with lower levels of diffusion are likely to have higher levels of sensitivity and specificity. The third measure was termed ‘Discrimination’ and defined as the average distance between the poles of a dipole as represented by the atlas. Tests with higher levels of discrimination are likely to have higher levels of sensitivity and specificity. A limitation of these measures is that they are only relevant if the research approach is predicated on the use of the atlas.

### Results

**Fig 8:**
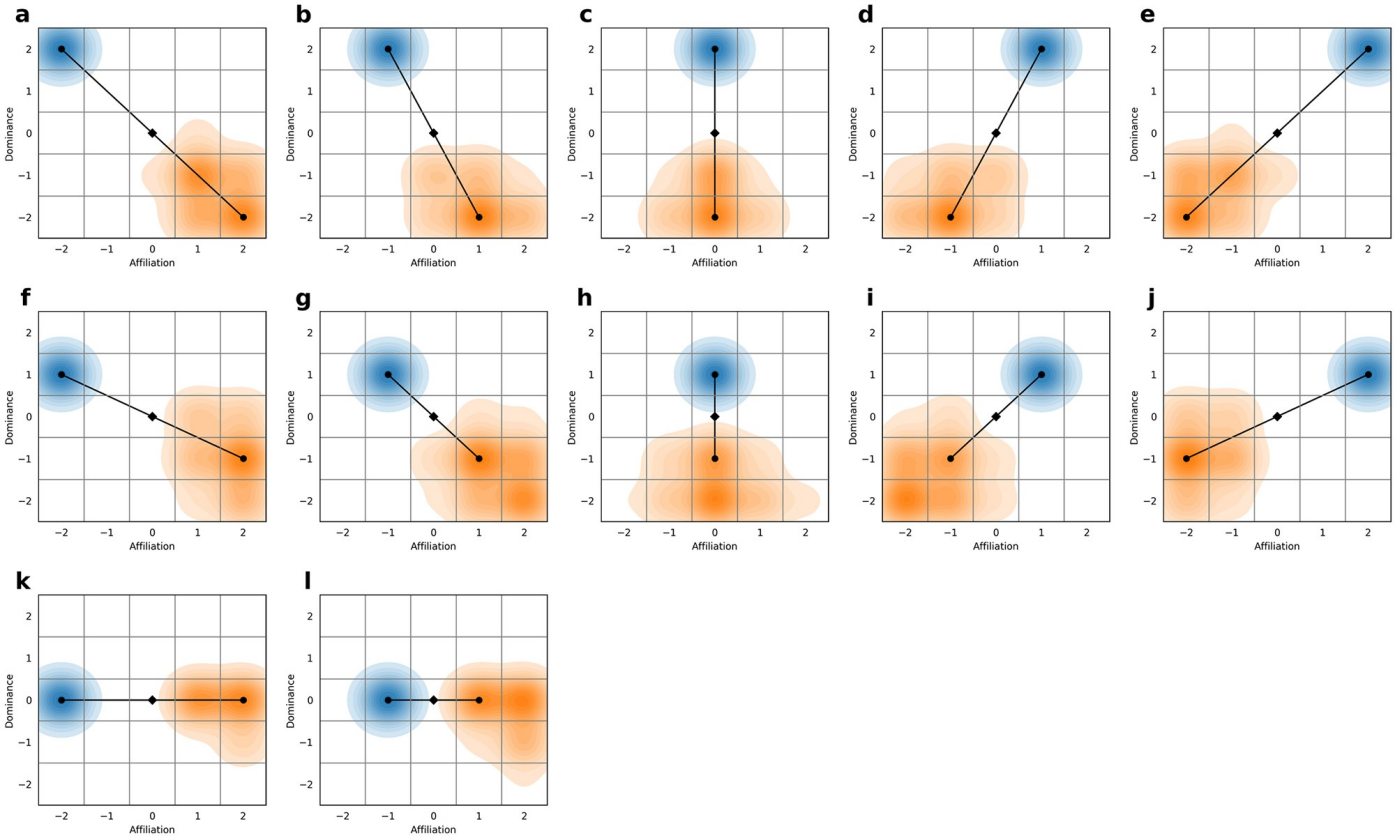
Binary pairs maximising overall contrast of the psychological test. For each of the 12 graphs, the blue and orange kernel density plots represent each side of the antonymic dipole. The kernel density plots are representative of the 299 observations (99.999999th percentile) out of 4 billion simulations that average distance between the 12 pairs, maximising the overall contrast of the psychological test. The lines shown on each graph represent the binary pairs that have a sufficient number of antonyms identified in the reference thesauri to allow the construction of a psychological test. The diamond at location (0,0) represents the point of reflection about which the antonymic pairs are reflected.

In total, 4 billion simulations were run, revealing that the average distance between antonymic pairs was maximised when antonymic pairs were selected from opposite sides of the matrix and reflected through the origin (Fig 8). For example, the selection of antonymic word pairs such as blissful (2,2) and despondent (-2,-2) have maximal contrast, and are located on opposite sides of the matrix (Fig 8E). The catalogue contained approximately 3,400 antonymic adjectival word pairs that were maximally contrasting. The alpha-beta pruning refinement revealed that 16 of the 10^23^ permutations maximised the average distance between the possible antonymic binary pairs (see Fig 8). Of these 16 combinations, only one had sufficient antonymic word pairs catalogued to facilitate a psychological test (Fig 8). Therefore, this combination was selected as the basis of the new psychological test. The simulation confirmed that the centre cell (0,0) is theoretically inappropriate for inclusion in the test, as it has no maximally distant pairing. Additionally, there were few antonymic pairs with an endpoint at (0,0).

The psychological test derived from the atlas was compared with pre-existing tests and the results are shown in Table 5.

**Table 5:** Facets of psychological test efficacy for several psychological tests and constructs.

Construct	Diffusion	Discrimination	Completeness	Dipoles
Criminal behaviours	47%	N/A	4%	0
DSM Schizophrenia	61%	0.0	4%	0
DSM Conduct	66%	0.0	4%	0
Dark Triad	33%	N/A	13%	0
DSM Autism Average	63%	1.4	8%	1
DSM Bipolar Average	78%	2.1	8%	1
Lead/Follow	68%	1.1	8%	1
Leadership	74%	1.8	8%	1
Ethical/Unethical	53%	3.3	8%	1
IPIP NEO	64%	1.6	42%	5
Atlas	0%	3.9	100%	12

A 12 question test based on the antonyms using the identified antonyms pairs is demonstrated in Table 6. Complete coverage of the atlas is obtained by selecting words located in cells from opposite sides of the atlas. This test may be used as either self-report or observer-report. S1 File lists the antonymic word pairs from which the 12 questions in Table 6 were selected. S2 Table provides an example of a personality (adjective) and emotion (abstract noun) questionnaires with 48 questions.

**Table 6:** Example 12 question test based on the atlas.

ID	Word				Antonym		Cells
1	despondent	▢	n/a	▢	hopeful	▢	(-2,-2)—(2,2)
2	sad	▢	n/a	▢	happy	▢	(-2,-1)—(2,1)
3	uncooperative	▢	n/a	▢	cooperative	▢	(-2,0)—(2,0)
4	disagreeable	▢	n/a	▢	agreeable	▢	(-2,1)—(2,-1)
5	unkind	▢	n/a	▢	kind	▢	(-2,2)—(2,-2)
6	cowardly	▢	n/a	▢	courageous	▢	(-1,-2)—(1,2)
7	discontent	▢	n/a	▢	content	▢	(-1,-1)—(1,1)
8	untalkative	▢	n/a	▢	talkative	▢	(-1,0)—(1,0)
9	unhelpful	▢	n/a	▢	helpful	▢	(-1,1)—(1,-1)
10	selfish	▢	n/a	▢	unselfish	▢	(-1,2)—(1,-2)
11	unambitious	▢	n/a	▢	ambitious	▢	(0,-2)—(0,2)
12	inactive	▢	n/a	▢	active	▢	(0,-1)—(0,1)

### Discussion

It has been demonstrated that for a psychological taxonomy consisting of a square matrix of 25 cells, the minimum number of questions required for a comprehensive psychological test is 12. It has also been shown that a psychological test consisting of antonymic dipoles taken from opposite sides of the atlas, when reflected about the origin, minimises test diffusion and maximises test discrimination and completeness. Study 2 derived a list of 1,620 antonymic dipoles suitable for such a psychological test (see S1 File). Examples of questions constructed from these antonymic word pairs include: ‘Are you generally friendly or unfriendly?’, ‘Are you generally kind or unkind?’, and, ‘Are you generally anxious or calm?’. Such questions are unambiguous and succinct.

Whilst such a test would be highly efficient, it is likely that a survey limited to 12 questions will have insufficient statistical power required for discriminatory testing. To achieve the requisite level of statistical power, it is suggested that multiple iterations of the 12 questions will be required (as in S2 Table). Future empirical studies will be required to determine the number of iterations required to achieve the level of statistical power suitable for particular applications. The atlas facilitated the development of a new form of psychological test that appears more comprehensive and effective than previous tests. However, a major limitation of this proposed test is that it has not been subject to normative testing and that the atlas taxonomy requires further validation.

## General discussion

The need for a parsimonious taxonomy of human personality, emotion and behaviour has been repeatedly identified[2,3,11] and is satisfied by the proposed atlas. The dimensions of the taxonomy, affiliation and dominance, have theoretical foundations in neurobiology, zoology and evolution[62–69]. A wide range of existing psychological and social taxonomies and constructs have been quantitatively visualised in these two dimensions. When visualised, the components of these taxonomies and constructs are clearly distinguished. The proposed psychological test, if validated, is likely to have advantages compared to previous psychological tests.

The atlas provides a theoretical basis upon which to redefine concepts such as emotional and social intelligence. Emotional intelligence might now be understood to mean an individual’s self-awareness of their emotional state relative to the atlas, and the subsequent ability to favourably alter their emotions. Similarly, it is hypothesised that social intelligence may newly be interpreted as the ability to accurately perceive one’s own and other’s behaviour relative to their positions within the atlas, to demonstrate competence in a wide range of social behaviours, and effectively navigate social interactions according to the atlas.

Cattell sought a periodic table. The atlas bears similarity to a periodic table; however, the dimensions are continuous, and is therefore best classified as an atlas. The atlas has the capacity for multilingual validation of this continuum. In summary, the present research has introduced a common language by which the linguistic associations between personality, emotion and behaviour can be understood, facilitating imperative future research into their causal relationships. A new and internet accessible catalogue of words is presented, substantially larger than previous catalogues, inclusive of verbs and nouns as well as adjectives. This two-dimensional atlas has been demonstrated to be theoretically based and offers empirical advantages over other taxonomies. The advantages of the atlas include its parsimony and unique ability to visualise psychological and social constructs in two dimensions. The applications of these findings extend to translational applications in clinical testing, workplace selection, social and emotional education, and research in the fields of sociology and psychology. Philosophically, this represents a significant step forward in how we might better understand both ourselves and others.

## Supporting information

S1 Table Atlas of personality, emotion and behaviour poster. Expanded version of Table 4 with 25 words for each combination of affiliation, dominance and domain.
(PDF)

S2 Table Example questionnaires with 48 questions. Two 48 Question tests. The first test uses antonymic adjectival descriptors of personality traits. The second using antonymic abstract noun descriptors of emotion.
(PDF)

S1 File Antonymic word pairs. Pool of antonymic word pairs. Groups of 12 may be selected to form a questionnaire based on the atlas.
(XLSX)
