# Supplementary material for: An atlas of personality, emotion and behaviour
Source: PLoS One. 2020 Jan 21;15(1):e0227877. doi: 10.1371/journal.pone.0227877 (PMC6974095; doi:10.1371/journal.pone.0227877)
Supplement: S1 Table — Expanded version of Table 4 with 25 words for each combination of affiliation, dominance and domain. (PDF) [file pone.0227877.s001.pdf]

Atlas of Personality, Emotion and Behaviour

	Affiliation														
	**-2**			**-1**			**0**			**1**			**2**		
**Dominance**	**Emotion**	**Behaviour**	**Personality**	**Emotion**	**Behaviour**	**Personality**	**Emotion**	**Behaviour**	**Personality**	**Emotion**	**Behaviour**	**Personality**	**Emotion**	**Behaviour**	**Personality**
**2**	aggression	abuse	atrocious	arrogance	berate	arrogant	braininess	begin	ace	backbone	administer	a1	ecstasy	allure	attractive
	barbarity	attack	cold-blooded	complacency	boast	demanding	brilliance	bolt	alert	boldness	advance	audacious	elation	appeal	awesome
	brutality	bash	corrupt	conceit	browbeat	extravagant	cleverness	compose	bright	bravery	assign	bold	enchantment	arouse	beautiful
	cruelty	beat	criminal	defiance	bulldoze	greedy	compulsion	create	brilliant	chivalry	boss	bonny	euphoria	attract	captivating
	depravity	belt	cruel	disobedience	bully	haughty	curiousness	dash	clever	courage	captain	brave	exhilaration	bewitch	charming
	enmity	blast	evil	ego	castigate	imperious	dedication	debate	curious	courageousness	command	choice	exuberance	captivate	dazzling
	fury	bomb	fierce	egotism	censure	intractable	drive	design	determined	dauntlessness	conduct	crackerjack	exultation	charm	engaging
	hate	burn	grim	envy	chastise	lofty	dynamism	discover	energetic	doughtiness	control	daring	fascination	dazzle	entertaining
	hostility	crush	hard	extravagance	coerce	lordly	energy	establish	exceptional	fearlessness	govern	direct	glory	enchant	excellent
	immorality	damage	immoral	greed	defy	obdurate	enterprise	hurry	fast	fortitude	guide	fine	grandeur	enrapture	fabulous
	indignation	defeat	inhuman	haughtiness	demand	obstinate	enthusiasm	improve	intellectual	gallantry	initiate	first-class	high	entertain	fantastic
	inhumanity	destroy	monster	insubordination	dictate	pompous	excitement	inaugurate	intelligent	grit	lead	first-rate	hope	enthral	glamorous
	iniquity	dispatch	monstrous	loftiness	intimidate	pretentious	fruitfulness	instigate	keen	gutsiness	manage	gallant	intoxication	entice	gorgeous
	meanness	fraud	perverse	need	lambaste	proud	imagination	institute	lively	heroism	marshal	grand	magnificence	excite	hopeful
	offence	hit	rotten	pomposity	lecture	rigid	initiative	interpret	outstanding	independence	mediate	great	majesty	fascinate	magic
	outrage	kill	savage	pompousness	press	stiff	intelligence	launch	professional	intestinal fortitude	organize	heavenly	merriment	incite	marvellous
	perversion	pound	severe	pretentiousness	punish	strict	intensity	master	proficient	intrepidity	overlook	high-class	paradise	induce	noble
	rage	rout	terrible	pride	rant	stubborn	invulnerability	mastermind	quick	mettle	oversee	nifty	perfection	inspire	perfect
	treachery	shoot	tough	self-admiration	rebel	unbending	keenness	originate	serious	moxie	referee	quality	radiance	lure	popular
	unkindness	smash	unsympathetic	self-importance	rebuke	uncompromising	liveliness	pioneer	sharp	nerve	regulate	remarkable	rapture	motivate	positive
	viciousness	strike	vicious	selfishness	reprimand	uncontrollable	tenacity	produce	smart	pluck	rule	slick	relish	rouse	splendid
	villainy	thrash	vile	smugness	reprove	unruly	vigor	pursue	superior	prowess	steer	strong	resplendence	seduce	superb
	violence	torment	violent	superiority	resist	unyielding	vigour	speed	vigorous	spine	steward	top	splendor	stimulate	terrific
	wickedness	whack	wicked	vainglory	scold	vain	vitality	start	visionary	spunk	superintend	top-notch	thrill	success	vivacious
	wrath	whip	wrong	vanity	suppress	wilful	zest	think	vital	strength	supervise	tremendous	triumph	tempt	wonderful
**1**	acrimony	clash	bitter	anger	agitate	aggressive	ability	act	active	amusement	advise	assured	blessedness	caper	animated
	animosity	degenerate	deplorable	annoyance	annoy	bastard	accomplishment	action	brisk	aplomb	advocate	big	bliss	carouse	blithe
	antagonism	degrade	disagreeable	artfulness	argue	bent	ambition	catch	busy	assurance	arrange	calm	buoyancy	cavort	bouncy
	antipathy	denigrate	disgusting	audacity	bar	choleric	aspiration	check	competent	belief	brief	certain	cheerfulness	celebrate	breezy
	aversion	denounce	distasteful	bother	beguile	crabby	capability	consider	conspicuous	calmness	contract	complete	cheeriness	cheer	bubbly
	bitterness	depreciate	dreadful	cheek	bind	cranky	capacity	determine	deep	comfort	counsel	composed	delight	dance	buoyant
	coarseness	diminish	false	competition	block	critical	competence	develop	diligent	composure	deal	confident	ebullience	enjoy	cheerful
	contempt	dismiss	filthy	craftiness	bluff	cross	competency	examine	dynamic	confidence	employ	content	felicity	escapade	cheery
	corruption	disregard	foul	dishonesty	bug	cunning	criticism	find	eager	conviction	engage	correct	festivity	exult	delighted
	debauchery	disrespect	ghastly	displeasure	challenge	deceitful	desire	fix	enthusiastic	decision	enjoin	definite	fun	feast	delightful
	degeneracy	infuriate	gross	duplicity	cheat	deceptive	determination	form	exact	dignity	influence	dispassionate	gaiety	fling	elated
	disdain	malign	horrible	frustration	conflict	dishonest	diligence	hasten	excited	ease	inform	easy	gladness	frolic	exuberant
	disgust	mock	hostile	guile	confound	dodgy	direction	make	firm	elegance	instruct	easy-going	glee	giggle	glad
	dislike	obstruct	mean	impatience	disagreement	fractious	efficiency	notice	fresh	enjoyment	negotiate	effective	gleefulness	invite	happy
	disloyalty	offend	nasty	impertinence	discredit	grumpy	inclination	plan	knowing	equanimity	offer	forward	happiness	jest	hearty
	grudge	reproach	noxious	impudence	dispute	impatient	industry	push	mighty	equilibrium	pose	innocent	hilarity	joke	high-spirited
	indecency	repulse	obscene	indiscretion	disturb	inflexible	intent	race	persistent	fitness	present	peaceful	jocundity	jump	jolly
	loathing	scorn	odious	insolence	fight	irresponsible	interest	resolve	profound	liberty	prompt	ready	jollity	lark	jovial
	malice	shun	offensive	irritability	frustrate	irritable	organization	review	progressive	peace	propose	secure	joviality	laugh	joyful
	nastiness	slander	repugnant	irritation	hassle	prickly	productiveness	rise	purposeful	resolution	recruit	sure	joy	party	lovely
	obscenity	spoil	repulsive	opposition	hustle	shady	proficiency	run	robust	satisfaction	relate	tranquil	joyfulness	play	merry
	resentment	taint	rude	presumption	irritate	tactless	purpose	study	significant	security	solicit	untroubled	joyousness	rejoice	peppy
	spite	vilify	tasteless	rudeness	quarrel	touchy	purposefulness	survey	snappy	serenity	suggest	unwavering	levity	revel	radiant
	vexation	vitiate	unpleasant	suspicion	reject	tricky	skill	view	sprightly	tranquillity	tell	well	rejoicing	romp	sunny
	vulgarity	weaken	vulgar	want	trick	wild	will	work	thorough	understanding	urge	worthy	revelry	sing	up
**0**	absurdity	break-up	absurd	blur	abate	awkward	appetite	be	alive	accuracy	answer	ardent	accord	ally	astute
	chaos	defect	asinine	complication	avoid	blank	breath	breathe	automated	attention	approach	careful	acumen	amalgamate	canny
	confusion	detach	cold	danger	cease	boring	consciousness	detect	automatic	attentiveness	assemble	clean	adroitness	associate	constant
	craziness	die	crackpot	difficulty	confuse	casual	heartbeat	digest	cognizant	balance	attend	clear	affiliation	attach	cooperative
	daftness	disappear	crazy	disruption	depart	confused	hunger	eat	conscious	carefulness	communicate	common	affinity	bond	cordial
	delirium	disconnect	cuckoo	distraction	desert	dirty	inner_self	exist	human	comprehension	comprehend	decent	alliance	breed	discerning
	detachment	disengage	daft	disturbance	displace	distant	innermost_self	feel	human_being	conformity	conform	equal	association	collaborate	emotional
	disaccord	dissociate	foolish	equivocation	dodge	dizzy	life	hear	mammal	congruence	endure	familiar	brotherhood	combine	ethical
	disharmony	dissolve	individual	fog	dwindle	eccentric	pulse	inhale	rhythmic	constancy	express	natural	closeness	conglomerate	fair
	disorder	distance	insane	haze	end	erratic	self	live	sentient	decorum	free	neat	community	connect	friendly
	distrust	disunion	kooky	headache	fade	fatuous	subconsciousness	look	subconscious	dexterity	gain	open	connection	consolidate	genial
	disunity	disunite	lunatic	impropriety	fail	freak	thirst	puff	vertebrate	discernment	gather	ordinary	family	cooperate	judgement
	doom	divide	mad	inconsistency	finish	funny		reside		equity	hold	proper	fellowship	coordinate	just
	folly	divorce	mindless	inconvenience	halt	indecent		rest		familiarity	indicate	reasonable	harmony	engagement	mature
	foolery	exit	nutty	injustice	ignore	indifferent		see		freedom	maintain	regular	insight	enter	passionate
	foolishness	isolate	outlandish	instability	lapse	irregular		sense		justice	meet	respectable	order	group	perceptive
	hallucination	perish	psychotic	interruption	leave	obscure		sit		perception	observe	rigorous	partnership	identify	rational
	idiocy	retire	screwy	irregularity	muddle	odd		sleep		precision	perceive	routine	passion	incorporate	romantic
	inanity	separate	senseless	isolation	neglect	opaque		smell		probity	return	sober	responsibility	join	savvy
	insanity	separation	silly	negligence	pass	peculiar		subsist		propriety	reveal	solid	shrewdness	liaison	sensible
	lunacy	sever	unfriendly	obscurity	question	remote		survive		shelter	say	steady	subtlety	link	shrewd
	madness	split	unsound	peculiarity	quit	strange		taste		simplicity	speak	straight	trust	merge	sincere
	nonsense	vacate	wacky	perplexity	shrink	unbalanced		touch		sobriety	stay	straightforward	union	rally	sophisticated
	senselessness	vanish	witless	preoccupation	terminate	unstable		utter		stability	talk	thoughtful	wisdom	reason	sound
	silliness	withdraw	zany	restraint	waste	vague		vocalize		transparency	understand	tidy	wit	unite	wise
**-1**	adversity	ache	blue	agitation	bellyache	agitated	disinclination	be_beaten	disinterested	acceptance	accept	accommodating	adhesion	appreciate	affable
	affliction	agonize	comfortless	alarm	bitch	anxious	disinterest	crawl	drab	agreement	accommodate	adaptable	admiration	boost	agreeable
	anguish	bawl	crestfallen	anxiety	bleat	diffident	doziness	dawdle	exhausted	appreciation	agree	considered	affection	contribute	amiable
	blues	bellow	dejected	apprehension	complain	doubtful	greenness	delay	flat	approval	aid	disciplined	agreeableness	credit	appropriate
	calamity	caterwaul	depressed	concern	crab	edgy	heaviness	dillydally	green	candour	allow	flexible	amity	cultivate	benign
	dejection	cry	disconsolate	deficiency	fluster	incorrect	idleness	disorganization	humdrum	courtesy	approve	forbearing	attachment	embrace	congenial
	disappointment	deplore	doleful	discomfort	fret	indefinite	immaturity	dither	idle	discretion	assent	guileless	civility	endorse	dedicated
	dispiritedness	flop	down	discontent	fuss	ineffective	inability	doze	ignorant	fidelity	assist	helpful	commitment	extol	dependable
	doldrums	glare	downcast	disquiet	gripe	ineffectual	incapability	drag	inactive	flexibility	assuage	honest	consolation	favour	faithful
	dolefulness	grieve	downhearted	distress	grizzle	insecure	incapacity	inaction	incompetent	forbearance	certify	ingenuous	courteousness	follow	genteel
	downheartedness	groan	dreary	doubt	grouch	jumpy	incompetence	inactivity	inept	forthrightness	confirm	law-abiding	decency	forgive	gracious
	forlornness	howl	forlorn	embarrassment	grouse	nervous	incompetency	kowtow	insipid	grace	consent	mannered	faithfulness	foster	honourable
	gloominess	lament	gloomy	fear	growl	nervy	indolence	laze	jaded	guilelessness	deliver	non-partisan	fealty	harmonize	liberal
	glumness	moan	glum	foreboding	grumble	restive	inexperience	loaf	lackadaisical	honesty	encourage	obliging	gentility	help	nice
	grief	mourn	heartbroken	fright	grump	restless	lassitude	loiter	lazy	lenience	give	ok	goodwill	honour	pleasant
	heartache	rue	joyless	hesitancy	mope	subdued	laziness	lounge	mediocre	leniency	grant	patient	graciousness	nourish	polished
	heartbreak	scream	lonely	hesitation	murmur	tense	lifelessness	postpone	pedestrian	liberality	permit	peaceable	gratitude	please	polite
	misery	snivel	lugubrious	inadequacy	mutter	troubled	naivety	relax	simple	patience	recognize	responsible	integrity	promote	refined
	pain	sob	miserable	indignity	nag	uncertain	resignation	shilly-shally	stale	piousness	relent	tame	like	reconcile	reliable
	regret	trembling	mournful	inhibition	niggle	uncomfortable	sleepiness	slacken	tired	pleasure	relieve	temperate	loyalty	refer	steadfast
	rejection	wail	sad	lack	repine	uneasy	slowness	slumber	unguarded	politeness	satisfy	unbiased	magnanimity	regard	sweet
	sadness	weep	sorrowful	trepidation	ruminate	unsettled	somnolence	snooze	uninspired	sincerity	settle	uncritical	morality	respect	true
	sorrow	whimper	sorry	trouble	vex	unsure	tiredness	stall	unproductive	softness	soothe	unhurried	purity	validate	trustworthy
	suffering	yell	sullen	uncertainty	whine	upset	torpor	unwind	unthinking	toleration	uphold	unsuspecting	sensitivity	value	warm
	unhappiness	yowl	unhappy	unease	worry	worried	weariness	wait	weary	willingness	vindicate	willing	tolerance	welcome	wholesome
**-2**	agony	break_down	alarmist	cowardice	abscond	afraid	apathy	atrophy	apathetic	biddability	authorization	acquiescent	adoration	acclaim	beneficent
	darkness	buckle	bleak	debilitation	blanch	beaten	boredom	crumble	birdbrained	compliance	authorize	amenable	allegiance	admire	benevolent
	defeatism	cave-in	defeatist	debility	blench	chicken	brainlessness	dally	bland	compliancy	bow	compliant	altruism	adore	bountiful
	depression	cave_in	desolate	dependence	cower	cowardly	disinterestedness	dawdling	bonehead	deference	capitulate	deferential	benevolence	adulation	caring
	desolation	collapse	despairing	dismay	cowering	effete	dullness	decay	brainless	demureness	commit	discreet	charity	baby	charitable
	despair	crumple	despondent	enervation	cringe	fearful	exhaustion	decline	dim	dependency	comply	docile	clemency	bless	compassionate
	desperation	deflate	dismal	enfeeblement	crouch	feeble	fatigue	deteriorate	dolt	docility	excuse	humble	compassion	cherish	devoted
	despondency	despond	doomsayer	faintness	decamp	fragile	fruitlessness	dillydallying	doltish	duty	favor	malleable	devotion	commend	feeling
	devastation	disintegrate	doomster	fearfulness	escape	impecunious	hebetude	droop	dope	frugality	flatter	meek	exaltation	compliment	fervent
	disaster	fall_apart	fatalistic	feebleness	flee	indigent	incuriousness	falter	dopey	humbleness	fulfil	moderate	fondness	deify	generous
	dread	fall_flat	hopeless	fragility	flinch	inelegant	ineptitude	give_in	dull	humility	guarantee	modest	generosity	esteem	gentle
	emptiness	fall_to_pieces	horror-stricken	frailness	recoil	needy	inertia	go_downhill	dumb	meekness	obey	obedient	gentleness	exalt	humane
	failure	fragment	incapacitated	frailty	retreat	pathetic	languor	goof	dunce	modesty	oblige	passive	humanity	glorify	indulgent
	fatalism	implode	insolvency	imperfection	run away	scared	lethargy	lag	idiot	obedience	pardon	pliable	kindliness	hail	kind
	gloom	panic attack	melancholic	impotence	scarper	shy	listlessness	languish	imbecile	passivity	provide	pliant	kindness	homage	kindly
	hell	self-destruct	morbid	inferiority	scram	spineless	malaise	regress	lamebrain	piety	release	quiet	love	indulge	lawful
	hopelessness	self-destruction	morose	infirmity	skedaddle	spiritless	monotony	rot	languid	pliability	sanction	slavish	mercy	laud	loving
	horror	self-murder	negative	intimidation	squinch	terrified	nonchalance	sink	oaf	respectfulness	serve	submissive	mildness	nurse	mild
	melancholy	self-slaughter	pessimist	languidness	squirm	timid	powerlessness	slack	oafish	self-sacrifice	submit	subservient	pity	nurture	righteous
	panic	slump	recession	oppression	take_off	timorous	sluggishness	subside	simpleton	servility	succour	tractable	reverence	praise	safe
	pessimism	suicide	tragic	spinelessness	tremble	toady	stagnation	succumb	slow	submission	supply	unassuming	righteousness	repair	sensitive
	ruin	thoughts of death	unfulfilled	threat	truckle	undistinguished	stupidity	vegetate	stupid	submissiveness	support	unobtrusive	sympathy	revere	soft
	terror	topple	woebegone	timidity	unnerve	unremarkable	susceptibility	waste_away	thick	subordination	sustain	unpretentious	tenderness	treasure	sympathetic
	woe	tumble	woeful	weakness	vamoose	wimpish	tedium	wilt	unintelligent	subservience	warrant	unselfish	virtue	venerate	tender
	wretchedness	unravel	wretched	wimpiness	wince	yellow	vulnerability	wither	vacuous	unselfishness	yield	yielding	warmth	worship	virtuous

Affiliation and Dominance Scale

	Affiliation	Dominance
**2**	The actor asserts, proffers, induces or predicts enduring affiliation with others	The actor asserts, proffers, induces or predicts enduring dominance over others
**1**	The actor asserts, proffers, induces or predicts transient affiliatiliation with others	The actor asserts, proffers, induces or predicts transient dominance over others
**0**	The actor's behaviour is neutral	The actor's behaviour is neutral
**-1**	The actor asserts, proffers, induces or predicts transient disaffiliation with others	The actor asserts, proffers, induces or predicts transient submission to others
**-2**	The actor asserts, proffers, induces or predicts enduring disaffiliation with others	The actor asserts, proffers, induces or predicts enduring submission to others

Category Usage

Category	Usage
**Emotion**	I feel a sense of ***emotion***; He/she is feeling a sense of ***emotion***
**Behaviour**	I ***behaviour*** her/him/them; They ***behaviour*** me/us
**Personality**	He/she/they are ***personality***; I am ***personality***

© 2020 Anthony E.D. Mobbs. Distributed under the terms of the Creative Commons Attribution License (CC BY 4.0)

https://doi.org/10.1371/journal.pone.0227877
