# Supplementary material for: An atlas of personality, emotion and behaviour
Source: PLoS One. 2020 Jan 21;15(1):e0227877. doi: 10.1371/journal.pone.0227877 (PMC6974095; doi:10.1371/journal.pone.0227877)
Supplement: S2 Table — Two 48 Question tests. The first test uses antonymic adjectival descriptors of personality traits. The second using antonymic abstract noun descriptors of emotion. (PDF) [file pone.0227877.s002.pdf]

Personality Trait (Adjective) Questionnaire

despondent	▢	neither	▢	hopeful	▢
negative	▢	neither	▢	positive	▢
pessimist	▢	neither	▢	optimist	▢
wretched	▢	neither	▢	excellent	▢
cheerless	▢	neither	▢	cheerful	▢
downbeat	▢	neither	▢	upbeat	▢
sad	▢	neither	▢	happy	▢
joyless	▢	neither	▢	joyful	▢
unfriendly	▢	neither	▢	friendly	▢
uncooperative	▢	neither	▢	cooperative	▢
solitary	▢	neither	▢	sociable	▢
untrusting	▢	neither	▢	trusting	▢
disagreeable	▢	neither	▢	agreeable	▢
intolerant	▢	neither	▢	tolerant	▢
ungrateful	▢	neither	▢	grateful	▢
unreliable	▢	neither	▢	reliable	▢
insensitive	▢	neither	▢	sensitive	▢
tough	▢	neither	▢	tender	▢
uncaring	▢	neither	▢	caring	▢
unkind	▢	neither	▢	kind	▢
afraid	▢	neither	▢	unafraid	▢
cowardly	▢	neither	▢	courageous	▢
indirect	▢	neither	▢	direct	▢
unadventurous	▢	neither	▢	adventurous	▢
agitated	▢	neither	▢	calm	▢
discontent	▢	neither	▢	content	▢
doubtful	▢	neither	▢	confident	▢
indecisive	▢	neither	▢	decisive	▢
inattentive	▢	neither	▢	attentive	▢
unrealistic	▢	neither	▢	realistic	▢
untalkative	▢	neither	▢	talkative	▢
inconsiderate	▢	neither	▢	considerate	▢
irresponsible	▢	neither	▢	responsible	▢
dishonest	▢	neither	▢	honest	▢
inflexible	▢	neither	▢	flexible	▢
unhelpful	▢	neither	▢	helpful	▢
arrogant	▢	neither	▢	humble	▢
demanding	▢	neither	▢	undemanding	▢
judgemental	▢	neither	▢	non-judgemental	▢
selfish	▢	neither	▢	unselfish	▢
unambitious	▢	neither	▢	ambitious	▢
unassertive	▢	neither	▢	assertive	▢
unenergetic	▢	neither	▢	energetic	▢
unmotivated	▢	neither	▢	motivated	▢
idle	▢	neither	▢	busy	▢
inactive	▢	neither	▢	active	▢
purposeless	▢	neither	▢	purposeful	▢
unproductive	▢	neither	▢	productive	▢

Emotion (Abstract Noun) Questionnaire

despair	▢	neither	▢	hope	▢
despondency	▢	neither	▢	jubilation	▢
gloom	▢	neither	▢	exuberance	▢
pessimism	▢	neither	▢	optimism	▢
cheerlessness	▢	neither	▢	cheerfulness	▢
misery	▢	neither	▢	bliss	▢
sorrow	▢	neither	▢	joy	▢
unhappiness	▢	neither	▢	happiness	▢
disconnection	▢	neither	▢	connection	▢
disharmony	▢	neither	▢	harmony	▢
disorder	▢	neither	▢	order	▢
unfairness	▢	neither	▢	fairness	▢
disagreeableness	▢	neither	▢	agreeableness	▢
disloyalty	▢	neither	▢	loyalty	▢
intolerance	▢	neither	▢	tolerance	▢
ungratefulness	▢	neither	▢	gratefulness	▢
callousness	▢	neither	▢	tenderness	▢
cruelty	▢	neither	▢	compassion	▢
hate	▢	neither	▢	love	▢
unkindness	▢	neither	▢	kindness	▢
cowardice	▢	neither	▢	bravery	▢
fearfulness	▢	neither	▢	fearlessness	▢
timidity	▢	neither	▢	boldness	▢
weakness	▢	neither	▢	strength	▢
discomfort	▢	neither	▢	comfort	▢
dissatisfaction	▢	neither	▢	satisfaction	▢
uncertainty	▢	neither	▢	assurance	▢
unease	▢	neither	▢	ease	▢
inequality	▢	neither	▢	equality	▢
instability	▢	neither	▢	stability	▢
unfamiliarity	▢	neither	▢	familiarity	▢
unreasonableness	▢	neither	▢	reasonableness	▢
disapproval	▢	neither	▢	approval	▢
displeasure	▢	neither	▢	pleasure	▢
impatience	▢	neither	▢	patience	▢
inflexibility	▢	neither	▢	flexibility	▢
disobedience	▢	neither	▢	obedience	▢
conceit	▢	neither	▢	modesty	▢
pride	▢	neither	▢	humility	▢
selfishness	▢	neither	▢	unselfishness	▢
apathy	▢	neither	▢	enthusiasm	▢
inertia	▢	neither	▢	drive	▢
lethargy	▢	neither	▢	energy	▢
vulnerability	▢	neither	▢	invulnerability	▢
disinterest	▢	neither	▢	interest	▢
incompetence	▢	neither	▢	competence	▢
purposelessness	▢	neither	▢	purposefulness	▢
unproductiveness	▢	neither	▢	productiveness	▢
